# Participant Experiences of Transcranial Direct Current Stimulation (tDCS) as a Treatment for Antipsychotic Medication Induced Weight Gain

**DOI:** 10.3389/fpsyg.2021.694203

**Published:** 2021-06-21

**Authors:** Luiza Grycuk, Francesca Moruzzi, Elena Bardjesteh, Fiona Gaughran, Iain C. Campbell, Ulrike Schmidt

**Affiliations:** ^1^Section of Eating Disorders, Department of Psychological Medicine, Institute of Psychiatry, Psychology & Neuroscience, King's College London, London, United Kingdom; ^2^Department of Psychosis Studies, Institute of Psychiatry, Psychology & Neuroscience, King's College London, London, United Kingdom; ^3^South London and Maudsley NHS Foundation Trust, Maudsley Hospital, London, United Kingdom

**Keywords:** schizophrenia, transcranial direct current stimulation, weight gain, tDCS, antipsychotic medication

## Abstract

**Background:** Despite the growing number of studies on the use of non-invasive brain stimulation in people with schizophrenia, there is limited research on participant views of such treatment methods.

**Aim:** Explore participant experiences and perceptions of transcranial direct current stimulation (tDCS).

**Methods:** Twelve people with schizophrenia took part in semi-structured interviews after having completed 5 sessions of tDCS. Thematic analysis was used to identify codes and themes.

**Results:** Five themes were identified: (1) motivation for study enrolment; (2) concerns about tDCS; (3) factors reducing the fear of tDCS; (4) experience of tDCS; (5) perceived effects of tDCS.

**Conclusions:** The study provides insight into the perceptions and experiences of each individual. Participants were concerned about the safety of tDCS and associated it with invasive procedures such as electroconvulsive therapy and lobotomy. Educational materials and a good relationship with the researcher played an important role in reducing the fear of brain stimulation. All participants described tDCS as uncomfortable, however, agreed that unpleasant sensations only lasted for a short while (20 s−5 min). After the first session, participants no longer felt anxious about the remaining ones. Strategies to improve treatment experience and study recruitment have been identified.

## Introduction

Non-invasive brain stimulation (NIBS) modalities, such as repetitive transcranial magnetic stimulation (rTMS) and transcranial direct current stimulation (tDCS), have been investigated as potential treatments for symptoms of schizophrenia, however, little is known about participants' views of such procedures. There is some support for the use of tDCS and rTMS in reducing auditory hallucinations in people with schizophrenia (Aleman et al., 2007; Freitas et al., 2009; Pond et al., 2017). A quantitative review of tDCS studies also showed small effect sizes for improvements in attention, working memory and cognitive ability (Mervis et al., 2017).

As of May 2021, there were 1,336 journal articles describing NIBS in people with schizophrenia and only one small study (*n* = 4) of rTMS for auditory hallucinations reported limited data on participant experience, with two individuals describing the stimulation as relaxing, and one as uncomfortable (Subramanian et al., 2013). There has been some interest in patient experience of NIBS, mainly rTMS, in other clinical populations (Walter et al., 2001; Rosedale et al., 2009; Mayer et al., 2012; Singh et al., 2018).

Participant experience of tDCS has been investigated in two studies. Tedesco Triccas et al. (2018) conducted interviews with 21 individuals with stroke who underwent tDCS combined with upper limb robot therapy. Participants mostly viewed tDCS as beneficial but found it unpleasant due to itching and burning sensations caused by the electric current. They hoped that in future a more comfortable delivery of tDCS would be developed. In addition, participants were concerned about the electrical current applied to the brain and safety of the intervention. Preliminary findings from participants with binge eating disorder who received tDCS highlighted similar concerns (G. Gordon, personal communication, January 20, 2021). Some participants associated tDCS with mental institutions and high voltage shocks, and some people found it “terrifying.”

The views of people about NIBS in general and tDCS specifically need further exploration. On the one hand, public perception may be affected by the overly optimistic media portrayals of tDCS as a painless and safe therapeutic device (Kekic et al., 2016). On the other hand, tDCS, which uses a weak electrical current of 1–2 milliamperes (mA) (Nitsche et al., 2008), could be confused with electroconvulsive therapy (ECT) which uses a current of 800–900 mA (Deng et al., 2011). tDCS may also be confused with deep brain stimulation, where electrodes are surgically implanted in the brain (Perlmutter and Mink, 2006). People with schizophrenia may be particularly vulnerable to such misapprehensions, especially if they have previously experienced compulsory or coercive treatment regimes. It is also unclear whether people experiencing positive symptoms affecting their sense of agency feel comfortable with a treatment that directly targets the brain.

Recent Medical Research Council guidance for evaluation of complex interventions in clinical trials recommends using qualitative methods to capture the experience of the intervention and improve understanding of the quantitative results (Moore et al., 2015). Additionally, it has been recommended to analyse such process data before the outcomes of the trial are known: this is to reduce bias in the interpretation of these data (Oakley et al., 2006). The present study used a qualitative approach to explore the perceptions and experiences of people with schizophrenia who received tDCS as treatment for antipsychotic medication induced weight gain. This investigation will complement findings from quantitative studies of NIBS and can aid the development of future studies and reduce barriers to establishing NIBS as a treatment.

## Materials and Methods

### Participants

The study was part of an ongoing feasibility randomised controlled trial (RCT) investigating the effects of tDCS and approach bias modification training (ABM) on food cravings in people with schizophrenia who take antipsychotic medication. Thirty people were recruited from the South London and Maudsley NHS Foundation Trust to participate in the RCT. Eligibility criteria included people aged 18–65 years, on a stable dose of antipsychotic medication and with a current diagnosis of schizophrenia or schizoaffective disorder. Diagnoses were made by the treating clinician according to the DSM-V criteria. During an initial telephone or in-person appointment with the researcher, the study was explained to potential participants. They also received an information sheet and a link to an educational video showing a typical tDCS session. In a second appointment with the researcher participants could discuss the materials and ask questions. Following enrolment, participants were randomly allocated to receive ABM with either real or sham tDCS. The full protocol is published elsewhere (Grycuk et al., 2020). Each participant attended 2 assessment and 5 treatment sessions and was reimbursed £90 for their time. A purposive sample of 12 participants was recruited from the RCT to take part in the qualitative interviews.

### Transcranial Direct Current Stimulation (tDCS)

TDCS was delivered at a current of 2 mA with the anode placed over the right dorsolateral prefrontal cortex (dlPFC) and the cathode over the left dlPFC. Current intensity of 2 mA has previously been used in the studies on food craving and binge eating (Kekic et al., 2014, 2017; Burgess et al., 2016). It is also the most commonly used setting in schizophrenia research (Kekic et al., 2016). In the real tDCS group, the current was delivered for the whole duration of the stimulation (20 min). In the sham (placebo) tDCS group, the current was automatically turned off after 30 s and for the remaining time a current pulse was delivered every 550 ms. This produced similar sensations to the real tDCS.

### Data Collection

Ethical approval for the study was obtained from the Oxford B Research Ethics Committee (ref: 19/SW/0095). Participant interviews took place in February-March 2020. They were conducted by two researchers (FM, EB) under the supervision of the main study researcher (LG) and lasted 10–30 min. Written consent was obtained before the interviews and participants were reimbursed £10 for their time.

All participants previously received 5 sessions of combined ABM and real or sham tDCS. Participants and the researchers were blinded to the group allocation during the interviews to reduce bias. Because the main focus of the study was on the experiences and perceptions of tDCS, and sham stimulation closely resembled the real one, data from both groups were analysed together. Participants were asked about their views and experiences of tDCS prior to and during the intervention, motivations for treatment and suggested improvements. A semi-structured interview format was used to allow for detailed descriptions. The topic guide was an adapted version of the topic guide previously developed by our group to explore patient experiences of rTMS (B. Dalton, personal communication, January 20, 2021).

### Analysis

Audio recordings were transcribed verbatim using oTranscribe (oTranscribe, n.d.) software and imported into NVivo 12. Braun and Clarke (2006) thematic analysis approach was used to search for patterns across the transcribed interviews. Responses were read repeatedly to familiarise the researchers with the data. Following this, initial codes were developed by assigning a brief descriptive label to each statement and from that, a coding framework was developed which was used to systematically code all transcripts. Codes were grouped into sub-themes and themes which were subsequently reviewed, refined, and named. This data analysis process was completed by researchers FM, EB, and LG. The final coding structure was validated by the remaining members of the research team. All researchers were blinded to the participant group allocation during data analysis and writing up of the results. Data for participants in the real and sham tDCS group were analysed together because the two types of stimulation produced similar sensations.

## Results

### Participant Characteristics

A purposive sample of twelve participants with a diagnosis of schizophrenia took part in the interviews. Demographics and clinical characteristics are shown in Table 1.

**Table 1:** Characteristics of the total sample (*n* = 12).

**Demographics**	
Age, mean (SD) years	44.7 (9.3)
Age, range, years	29–61
BMI, mean (SD), kg/m^2^	32.8 (6.9)
BMI, range, kg/m^2^	20.1–45.9
**Gender**, ***n*** **(%)**	
Male (P2, P3, P5, P6, P9, P11)	6 (50)
Female (P1, P4, P7, P8, P10, P12)	6 (50)
Illness duration, mean (SD) years	16 (11.5)
Illness duration range, years	2–43
**Ethnicity**, ***n*** **(%)**	
White	3 (25)
Black	5 (41.7)
Mixed	1 (8.3)
Asian	1 (8.3)
Other	2 (16.7)
**Education**, ***n*** **(%)**	
BA/BSc	4 (33.3)
College	4 (33.3)
Secondary	4 (33.3)
**Employment status**, ***n*** **(%)**	
Unemployed	9 (75)
Employed	3 (25)

### Key Themes

#### Reasons for Participation

Half of the participants hoped that their involvement would make a positive contribution to science and subsequently improve the lives of others: “I thought I would be helping my community” (P6). The same number of people (*n* = 6) affirmed their interest in psychological research and said they regularly sign up for studies which they meet the inclusion criteria for: “I'm happy to participate in any new research… because I want new treatments to be developed for schizophrenia and the problems that it has” (P5).

Three participants admitted that they enrolled on the study to make “a bit of extra money” (P2) and because they had free time and few commitments: “I don't work at the moment. All I do is go to Mind groups and things, so it was like that. And I thought I might as well do it, because I'm doing nothing else” (P10). Seven people hoped that tDCS would facilitate weight loss by reducing food cravings: “I was desperate. I just wanted to find out could the brain stimulation curb my appetite” (P1).

Several (*n* = 7) participants expressed confusion about the aims of the study. Some thought tDCS would help with “retaining information” (P7), “concentration” (P9) or to “reduce” or “replace antipsychotic medication” (P4). Furthermore, one person admitted that they “didn't know initially it was a brain stimulation” (P12) until they arrived at the first session, and another one felt they “didn't really understand the question” (P7) being researched.

#### Concerns About tDCS

Most of the participants (*n* = 9) experienced feelings of uneasiness about receiving brain stimulation. Their concerns can be grouped into three categories: (a) damage to the brain, (b) unexpected side effects, and (c) altered thinking or personality.

Participants feared that stimulation may cause harm to their brains: “Lobotomy-type, I was worried whether it would kill brain cells” (P4); “Something [could happen] to the brain that you don't know” (P7). One person recalled that they were “reassured” upon learning the stimulation uses a “minor electric current” (P1). Another participant pointed out that people may confuse tDCS with ECT:

“I don't have any real problems with the brain stimulation as a method… particularly these low current ones… people are concerned about it because of the therapy—I believe it's for depression… the high voltage ones…electric convulsive… I wouldn't want to go through that” (P5).

Some people were concerned about unexpected side effects because they did not consider tDCS as an established treatment: “I was worried about the medical implications and if there's any risk because this is research and you don't know the results” (P2). Correspondingly, because tDCS was in the research phase, people thought it could cause long-term side effects that were not yet known to the researchers:

“There is this case where they gave people this medication and they found out years later that it caused them harm, and I thought […] oh my god, what if something that's not been discovered [happens]. That'd be dangerous” (P8)

One participant mentioned that a friend of theirs was worried that tDCS would alter the participant's way of thinking. The participant herself was not concerned about this: “I didn't think it would put thoughts into my head or anything like that, because I know being schizophrenic you can think like that, but I didn't think anything like that” (P10). Another person who felt comfortable with receiving brain stimulation admitted that they did not inform any family members about their participation because they “did not want to worry them” (P1).

#### Factors Reducing the Fear of tDCS

The importance of communication with the researcher was highlighted by most of the participants (*n* = 9). They recalled that receiving information about study procedures, tDCS mechanisms of action and side effects reduced their pre-treatment anxiety. Participants reasoned that “research ethics committees [were] exerting proper controls over psychological studies” (P5), and “these types of studies are done all the time” (P12). One person mentioned that the educational video demonstrating a typical tDCS session was particularly helpful to them:

“I did think that maybe I didn't want to do it but when I found out about it. I was reassured that it would be okay because I saw a video explaining what happens and I realised that it's not a strong electric current it's a minor electric current” (P1).

A good relationship with the researcher played an important role in reducing the fear of tDCS. Participants said that they trusted the researcher because they were friendly, seemed experienced, and provided easy to understand information:

“The first time I've come I was a bit nervous. But she [the researcher] makes you feel so at ease. She's friendly and she explains everything really well. And I think, one of the reasons I wasn't that nervous was before I did this I had quite a few telephone conversations with [the researcher] and she's really good. She explains everything” (P10).

Some people reported that doing their own internet search on brain stimulation reduced their fears. For others, their curiosity about the novel treatment had a similar effect.

#### Experience of tDCS

All participants recalled the discomfort felt during the tDCS sessions. Its magnitude varied considerably, with some describing it as mild, being “itchy and warm” (P5), and others as painful, being “stinging and burning” (P4). One person remarked: “I haven't actually been on an electric chair before, but it felt like I was on a mini electric chair” (P6). In all cases discomfort was reported to be short-lived, lasting between 20 s and 5 min: “It only lasts for 20 s, and then it disappears. But you got to remember not to worry if it's stinging you” (P3).

Participants explained that sensations felt during the sessions were similar to those described by the researchers prior to the start of the study. They expected some discomfort and were not scared when they experienced it: “I was alright cause I knew that there might be some side effects” (P4). Two participants had expected the stimulation to be “more invasive and painful” (P4), whereas one person had not been “expecting it to sting as much as it did” (P11). Three people reported side effects such as “wheezing, tiredness” (P2) and “slight headaches” (P4, P12), however, they were not sure if these were caused by the brain stimulation.

Overall, after completing the first stimulation, participants reported no longer feeling anxious about the remaining sessions: “I was a bit anxious when I first started, because I didn't know what to expect really. It was very easy. Straightforward. Very little discomfort” (P10). The researcher administering the stimulation played an important role in participant experience by explaining the procedure and providing reassurance: “They talked you through it, help you understand what was going on and helped you to understand what the side effects could be, and you know, just made the experience a lot easier” (P4).

#### Perceived Effects of tDCS

Despite the discomfort, all participants experienced the treatment as positive and, in some cases, enjoyable: “I've really enjoyed [the treatment], I found it interesting” (P10). Participants mentioned feeling happier and having improved relaxation, motivation, and concentration following the sessions. Three people mentioned a renewed sense of confidence and optimism: “[The improved] confidence was good. But how does it affect my life? I'd already been thinking that it's helped me think [that] maybe there's ways I could improve my recovery more” (P8). Another person said: “I think it stirred up thoughts and emotions in me [that] were probably locked away” (P6), but did not elaborate on the change.

One third of the participants (*n* = 4) reported an increased awareness of “healthy eating” following the treatment: “It's made me feel a little better about what I'm eating, I think. It's made me wonder how much I eat junk food and because it's a habit I can change” (P8). However, the rest expressed scepticism towards treatment effectiveness. Out of these, one person (P10) believed they had received the sham stimulation.

There were very few suggestions on how the treatment could be improved. A few people hoped the electrode straps could be “more comfortable” (P4). Some suggested home visits, whereas others said they would not feel comfortable with the researchers coming to their homes. A couple hoped for fewer and shorter tDCS sessions. One person (P8) said they would have preferred more information on research background and mechanisms involved.

## Discussion

This study explored the perceptions and experiences of tDCS in people with schizophrenia. Five major themes emerged: motivation for study enrolment, concerns about tDCS, factors reducing the fear of tDCS, experience of tDCS, and perceived effects of tDCS.

In line with the specific aims of this study, more than half of the participants hoped for weight loss. Some said that they had struggled with antipsychotic medication induced weight gain for a long time and felt that there was no effective treatment for it. A lack of available treatment options was also the most commonly reported reason why people with depression (Clarke et al., 2018) and anorexia nervosa (B. Dalton, personal communication, January 20, 2021) were willing to try rTMS. These views were echoed in a study of eating disorder clinicians who considered NIBS as suitable mainly for patients with unsuccessful previous treatments (Dalton et al., 2020). Altruistic motives (i.e., contributing to the community, a wish to advance treatment research in schizophrenia) were also a common reason for participating in the study. This suggests that it is worth highlighting these aspects when recruiting research participants.

Most participants had fears and concerns about tDCS, prior to starting, e.g., stimulation killing brain cells and causing side effects. Importantly, none of the participants expressed anxiety over intentional harm or external interference with the brain with one person specifically mentioning that they did not think tDCS could cause thought insertion. Participant fears are consistent with those reported by other patient groups receiving NIBS [e.g., stroke rehabilitation (Tedesco Triccas et al., 2018), binge eating disorder (G. Gordon, personal communication, January 20, 2021), anorexia nervosa (B. Dalton, personal communication, January 20, 2021)].This suggests that the concerns reported here were not schizophrenia specific.

Factors reducing concerns about tDCS included the provision of accessible information about tDCS and study procedures and a good relationship with the researcher. However, it was only after completing the first session that participants reported no longer feeling anxious about the remaining ones. Experience of the first session seemed to be crucial in participants forming a considered opinion of tDCS and setting expectations of future sessions.

Given the intensity of some of the fears expressed, we recommend that NIBS therapists should possess strong interpersonal and communication skills to educate and engage participants. In particular, they need to be able to recognise, validate, and respond appropriately to any safety concerns expressed. For individuals who are especially anxious about receiving NIBS it may be beneficial to offer a “trial” session during which they can experience the stimulation. This may be particularly useful when NIBS is combined with cognitive tasks (as in our trial) to reduce the effects of participants' anxiety on task performance.

The experience of receiving tDCS was described as uncomfortable by all participants. Stimulation felt itchy, warm and in some cases burning. Most people agreed that these sensations were only transient and were not unduly distressed by them. There is some evidence to suggest that women report more discomfort than men during 2 mA tDCS stimulation (Workman et al., 2020). Gender differences could not be assessed in this study due to the qualitative nature of the data, but will be investigated as part of the feasibility RCT. It would be interesting to know if men report less discomfort than women because of the social desirability bias, i.e., because they do not want to be perceived as weak. Three individuals reported side effects such as wheezing, tiredness or a headache, but were not sure if these were caused by the stimulation. Accordingly, we suggest that future studies should pay attention to explaining common side effects of NIBS. It is also worth noting that the intensity of the stimulation influences the perception of sensations, with 2 mA intensity producing more discomfort that 1 mA (Fertonani et al., 2015). Therefore, experiences of people who received 1 mA stimulation may be different to those described in this study.

Despite discomfort participants completed all of their sessions. However, it is not clear to what extent this was influenced by the monetary compensation provided. In other tDCS studies too (stroke rehabilitation) participants also reported feelings of discomfort (Tedesco Triccas et al., 2018), whereas individuals receiving rTMS for depression did not raise such concerns (Walter et al., 2001; Mayer et al., 2012). RTMS was perceived as less frightening than “having something done at the dentist” by most adults (88%) (Walter et al., 2001) and less than half of adolescents (37.5%) (Mayer et al., 2012). This difference could be due to the fact that 2/3 of the adult sample in that study had previously had ECT (Walter et al., 2001), whereas the adolescents would have been more treatment naïve. These findings suggest that the degree of distress experienced during application of different NIBS may depend on previous treatment experiences.

Overall, all participants rated their experience of the treatment as positive. They found it difficult to think of how to improve this. It is not possible to be conclusive as to whether this was because they were genuinely satisfied with all elements of the treatment, or uncomfortable with expressing their opinions. In terms of efficacy of the treatment, one third of the participants reported an increased awareness of “healthy eating.” Some recalled beneficial effects of the stimulation such as improved confidence, motivation, and concentration, as well as feeling happier and more relaxed. Such effects on mood are in line with the current research on NIBS for depression, which targets the same brain area—dlPFC that was stimulated in this study. Reported benefits of tDCS, however, need to be interpreted with caution because it is not known how many of the participants received real or sham tDCS. Additionally, factors that may affect the efficacy of the stimulation, e.g., female hormones, neurotransmitters or cortical bone structure (Rudroff et al., 2020), were not taken into consideration.

Although great care was taken to explain the study procedures in detail and using different formats (images, video), understanding of the study varied considerably between participants. During the interview, several individuals reported feeling well-informed about the RCT and at the same time showed misunderstanding of the study aims, questioned the interviewer on the purpose of the study and the use of tDCS. We do not know whether participants originally understood the study aims but had forgotten them by the time of the interview, or whether they had not grasped them fully at the beginning. Confusion about study aims could potentially have resulted from cognitive symptoms of schizophrenia such as deficits in attention, working memory, verbal learning and memory, and executive functions (Fioravanti et al., 2012). To make study information more accessible and retainable, we recommend breaking it down into smaller portions and repeating it. A separate information sheet for family members would also be beneficial. Families are often involved in the treatment/care of individuals with schizophrenia and engaging them can increase the knowledge and acceptability of NIBS.

## Strengths

To our knowledge, this is the first study investigating perceptions and experiences of tDCS in people with schizophrenia. The study used a qualitative approach to allow for in-depth exploration of the topic.

## Limitations

Results are based on individuals who participated in an ongoing feasibility RCT and may not reflect the views of people who did not take part. The exact uptake of the intervention will be published as part of the RCT. An additional focus group or survey of people who did not want to receive tDCS would enhance the present findings, especially with respect to participants' concerns. Participants were paid for their participation in the RCT and the interview. It is not known if monetary incentive impacted the participants' views and acceptability of the treatment.

## Conclusions

This qualitative study explored the perceptions of people with schizophrenia who received tDCS as treatment for antipsychotic medication induced weight gain. Strategies to improve treatment experience and study recruitment have been identified. The findings highlight the importance of providing information on treatment safety, side effects, addressing common concerns, and ensuring a thorough understanding of research aims. Future studies should look at the experiences of other NIBS applications in this population.

## Data Availability Statement

The original contributions presented in the study are included in the article further inquiries can be directed to the corresponding author.

## Ethics Statement

The protocol version 1 dated 04/04/2019 was approved by the Oxford B Research Ethics Committee (REC, reference number: 19/SW/0095) and is a registered clinical trial under the International Standard Randomised Controlled Trial Number (ISRCTN) registry (registration number: ISRCTN13280178). The participants provided their written informed consent to participate in this study.

## Author Contributions

LG, FM, EB, FG, IC, and US made significant contributions to the design and drafting of this article. LG drafted the original manuscript, FG, IC, and US made revisions to this manuscript. US is the principal investigator of the study. All authors have approved the final manuscript and accept responsibility for the accuracy and integrity of this work.

## Conflict of Interest

The authors declare that the research was conducted in the absence of any commercial or financial relationships that could be construed as a potential conflict of interest.

## Abbreviations

tDCS: transcranial direct current stimulation
NIBS: non-invasive brain stimulation
rTMS: repetitive transcranial magnetic stimulation
ECT: electroconvulsive therapy
RCT: randomised controlled trial
ABM: approach bias modification training
dlPFC: dorsolateral prefrontal cortex.
